# Long-term local tumor control by endoscopic radiofrequency ablation for ampullary carcinoma with biliary extension in a patient with familial adenomatous polyposis

**DOI:** 10.1055/a-2764-4570

**Published:** 2026-01-20

**Authors:** Keita Fujisaki, Susumu Hijioka, Yoshikuni Nagashio, Shota Harai, Daiki Yamashige, Joshua Josef Torres, Takuji Okusaka

**Affiliations:** 1 13874 Department of Hepatobiliary and Pancreatic Oncology, National Cancer Center Japan, Tokyo, Japan; 2 Department of Internal Medicine, Silliman University Medical Center, Dumaguete City, Philippines

A Habib EndoHPB catheter (Boston Scientific) is a bipolar endoscopic radiofrequency ablation (RFA) device that enables circumferential ablation of the biliary tract 1 . While adjunctive endoscopic RFA has shown efficacy for malignant biliary strictures and as salvage treatment for remnant bile duct lesions after papillectomy 2 3 4 , reports of its use as initial therapy for ampullary carcinoma (AC) remain limited.

**Fig. 1:**
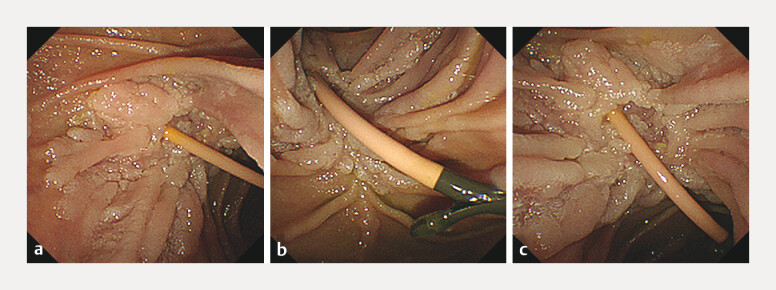
Duodenoscopic findings of the descending duodenum. **a** – **c** The ampulla appeared depressed with lateral extension into the adjacent surrounding duodenal mucosa.

**Fig. 2:**
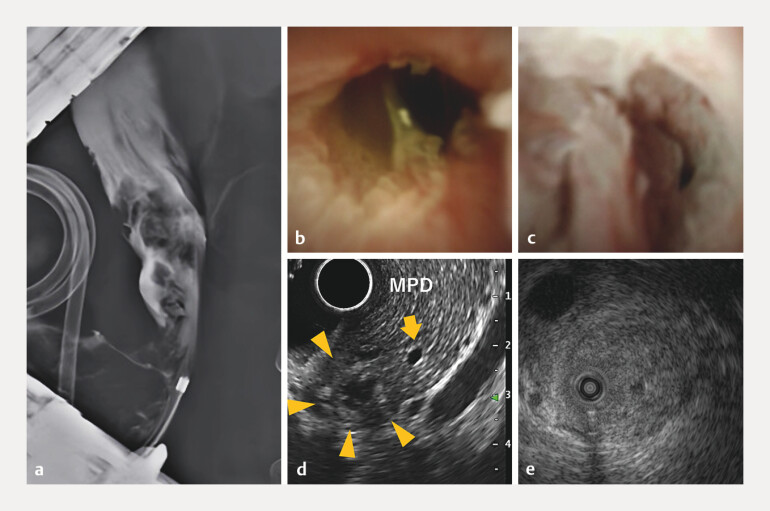
Pre-treatment biliary evaluation. **a** Endoscopic retrograde cholangiography (ERC) showing an irregular radiolucent area in the distal bile duct. **b** and **c** Peroral cholangioscopy revealing a papillary tumor. **d** An endoscopic ultrasound (EUS) image showing no evidence of pancreatic invasion. **e** Intraductal ultrasonography (IDUS) showing no invasion of the bile duct muscularis layer.

We report a surgically ineligible 48-year-old man with familial adenomatous polyposis who was diagnosed with intraepithelial AC (cT1aN0M0, Stage IA) with extension into the distal bile duct and duodenum. The patient had a history of five prior open abdominal surgeries, including total colectomy, and multiple intra-abdominal desmoid tumors. Duodenoscopy revealed a depressed ampulla with a laterally spreading whitish elevated lesion ( Fig. 1 **a–c** ), and endoscopic retrograde cholangiography and cholangioscopy demonstrated a papillary tumor extending into the distal bile duct ( Fig. 2 **a–c** ). Endoscopic ultrasound and IDUS demonstrated preservation of the bile duct muscularis and no pancreatic invasion ( Fig. 2 **d, e** ). Biopsies confirmed an adenoma in the duodenum and adenocarcinoma in the ampulla and intrapancreatic bile duct.

**Fig. 3:**
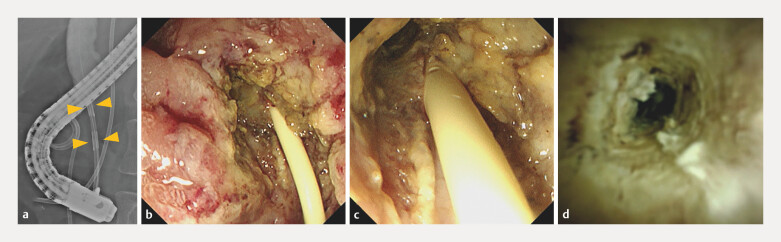
Images of RFA. **a** An ERC image showing ablation of the distal bile duct using the Habib EndoHPB catheter. **b** and **c** A duodenoscopic view after ablation showing circumferential ablation of the ampulla. **d** A cholangioscopic view confirming the circumferential ablation of the intraductal tumor. ERC, endoscopic retrograde cholangiography; RFA, radiofrequency ablation.

**Fig. 4:**
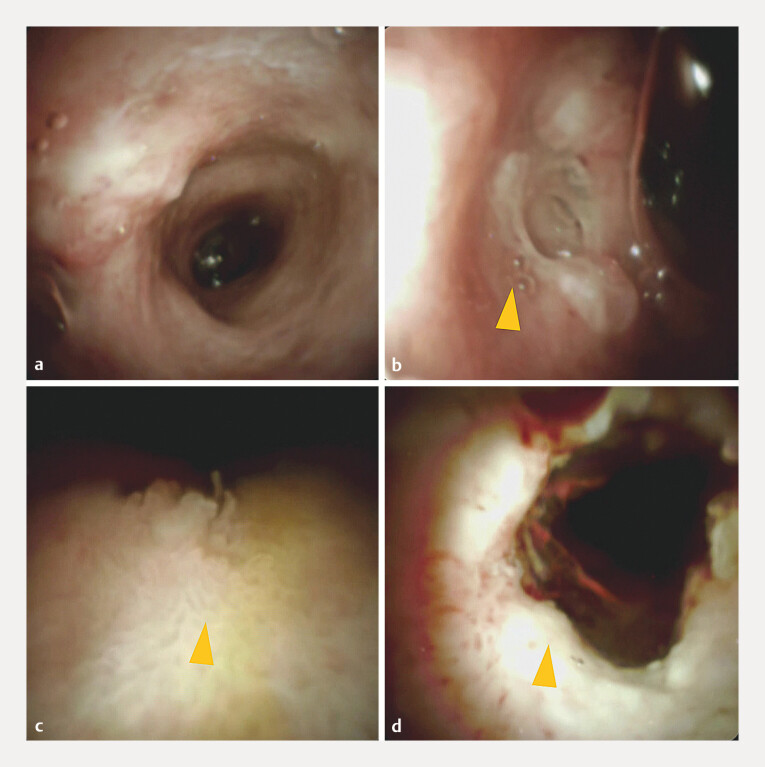
Cholangioscopic findings at 3 months post-RFA. **a** Confluence of the cystic duct. **b** Biopsy just below the cystic duct confluence revealed no malignancy. **c** Biopsy from the distal bile duct revealed no malignancy. **d** Biopsy from the area just above the ampulla revealed no malignancy. RFA, radiofrequency ablation.

**Fig. 5:**
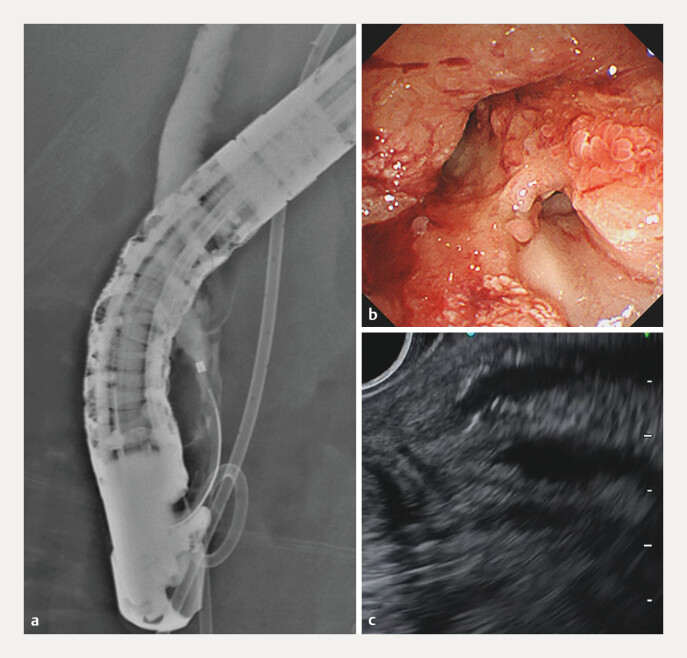
Endoscopic findings at 2 years post-RFA. **a** An ERC image showing no elevated lesions in the distal bile duct; biopsy revealed no malignancy. **b** A duodenoscopic view showing no suspicious findings at the orifice of the bile or pancreatic duct. **c** No tumor extension into the bile and pancreatic ducts was observed. ERC, endoscopic retrograde cholangiography; RFA, radiofrequency ablation.

Given the patient’s poor surgical candidacy, endoscopic RFA was performed following pancreatic duct stent placement. The Habib EndoHPB catheter was applied from the distal bile duct to the ampulla for 15–25 seconds each 5 , achieving complete tumor ablation, as confirmed by post-RFA endoscopy and cholangioscopy ( Fig. 3 **a–d** ). A covered self-expandable metal stent was then deployed to ensure biliary drainage. Endoscopic surveillance at 3 months ( Fig. 4 **a–d** ) and 2 years post-treatment ( Fig. 5 **a–c** ) revealed no local tumor recurrence or distant metastasis ( Video 1 ).

Endoscopic radiofrequency ablation for ampullary carcinoma with intraductal extension, showing complete tumor ablation and 2-year recurrence-free follow-up in a patient unsuitable for surgery.Video 1

This case suggests that endoscopic RFA may offer an alternative, particularly in cases like this, for surgically unfit patients with localized ampullary or biliary malignancies. Further studies are warranted to validate its role as a primary therapeutic option in selected cases.

Endoscopy_UCTN_Code_TTT_1AR_2AF
